# Non-typeable *Haemophilus influenzae* isolates from patients with chronic obstructive pulmonary disease contain new phase-variable *modA* methyltransferase alleles controlling phasevarions

**DOI:** 10.1038/s41598-019-52429-6

**Published:** 2019-11-04

**Authors:** John M. Atack, Timothy F. Murphy, Melinda M. Pettigrew, Kate L. Seib, Michael P. Jennings

**Affiliations:** 1 Institute for Glycomics, Griffith University, Gold Coast, Queensland 4222 Australia; 2 Clinical and Translational Research Center, University at Buffalo, State University of New York, 875 Ellicott Street, Buffalo, NY 14203 USA; 3 Yale School of Public Health, New Haven, CT USA

**Keywords:** Bacterial genetics, Pathogens

## Abstract

Phasevarions (phase-variable regulons) are emerging as an important area of bacterial gene regulation. Many bacterial pathogens contain phasevarions, with gene expression controlled by the phase-variable expression of DNA methyltransferases via epigenetic mechanisms. Non-typeable *Haemophilus influenzae* (NTHi) contains the phase-variable methyltransferase *modA*, of which multiple allelic variants exist (*modA1-21*). We have previously demonstrated 5 of 21 these *modA* alleles are overrepresented in NTHi strains isolated from children with middle ear infections. In this study we investigated the *modA* allele distribution in NTHi strains isolated from patients with chronic obstructive pulmonary disease, COPD. We demonstrate that the distribution of *modA* alleles in a large panel of COPD isolates is different to the distribution seen in middle ear infections, suggesting different *modA* alleles may provide distinct advantages in the differing niches of the middle ear and COPD airways. We also identified two new phase-variable *modA* alleles – *modA15* and *modA18* – and demonstrate that these alleles methylate distinct DNA sequences and control unique phasevarions. The *modA15* and *modA18* alleles have only been observed in COPD isolates, indicating that these two alleles may be markers for isolates likely to cause exacerbations of COPD.

## Introduction

Non-typeable *Haemophilus influenzae* (NTHi) is a major human-adapted pathogen that is the etiological agent of a number of acute and chronic diseases of the human respiratory tract^1,2^, and for invasive infections such as septicaemia and meningitis^3–5^. NTHi is intimately involved with the pathogenesis of chronic obstructive pulmonary disease (COPD). Approximately 65 million people worldwide suffer from COPD, which is the fourth most common cause of death globally^6^. NTHi is known to colonise patients with COPD and persist for extended periods of time. This results in increased inflammation and resulting tissue damage, and further loss of pulmonary function^7^. A sudden worsening of COPD symptoms is known as an exacerbation. Half of all COPD exacerbations are the result of bacterial infection, with NTHi being the most common bacterial pathogen isolated from COPD patients experiencing an exacerbation^8^. Individual NTHi strains show a high level of genotypic and phenotypic heterogeneity^9^. Previous work has shown that a significant number (33%) of COPD exacerbations occur upon acquiring a new strain of NTHi^10^, and that the lung microbiome changes both during and after COPD exacerbations^11^. There is also a demonstrated link to particular genomic features associated with strains of NTHi isolated from patients experiencing a COPD exacerbation^12^, which is strongly indicative that COPD exacerbations are not only due to host factors, but are triggered by bacterial factors.

We have previously reported the presence of phase-variable regulons (phasevarions)^13^ in a range of human adapted bacterial pathogens such as *Streptococcus pneumoniae*^14^, *Neisseria gonorrhoeae* and *Neisseria meningitidis*^15^, *Helicobacter pylori*^16^, *Haemophilus influenzae*^17^, and *Moraxella catarrhalis*^18^. Phasevarions result in differential expression of a regulon of genes via epigenetic mechanisms through the phase-variation (ON-OFF switching or variation in specificity) of DNA methyltransferases associated with restriction-modification (R-M) systems. In all cases described, phasevarions control the expression of genes required for pathobiology. In many cases phasevarion switching also leads to differential expression of vaccine candidates in multiple bacterial pathogens such as *S. pneumoniae*^14^, *N. gonorrhoeae* and *N. meningitidis*^15^, and NTHi^19^. The presence of phasevarions in several major human pathogens makes identification of stably expressed antigens problematic, as the genes subject to regulation by phasevarions do not contain any easily identifiable features. Currently, the only way to identify genes in a phasevarion is by detailed study of gene and/or protein expression in the alternate phasevarion states. The identification of surface antigens regulated by phasevarions is critical to find stably expressed vaccine antigens and also to understand their role in pathobiology.

In work carried out with a large panel of NTHi isolates taken from children with middle ear infection, or otitis media (OM), we have previously demonstrated that ~65% of these NTHi isolates contain one of just five phase-variable Type III N^6^ adenine DNA methyltransferases, ModA2, 4, 5, 9, or 10, among the 21 known *modA* alleles. Each *modA* allele methylates a distinct DNA sequence and controls a different set of genes, i.e., a different phasevarion^19^. The genes encoding these alleles are highly conserved at their 5′ and 3′ regions (95% DNA identity), but contain a highly variable central region^20^, encoding the Target Recognition Domain (TRD). The TRD dictates the sequence recognised and methylated by the ModA protein. A different TRD means a different sequence is methylated, and consequently a different phasevarion of regulated genes is controlled. We hypothesised that strains of NTHi isolated from a different host micro-environment, i.e., the airways of COPD patients, may contain a different set of *modA* alleles when compared to those isolated from the middle ear of children with OM^19^, as the environment of the COPD lung differs significantly from the OM middle ear. Here we describe the *modA* alleles present in a large collection (n = 269) of NTHi strains isolated from the sputum of people with COPD^21^ to investigate the *modA* allele distribution, and characterise gene expression differences resulting from phase-variation of two previous unstudied *modA* alleles, *modA15* and *modA18*.

## Methods

### Bacterial strains and growth conditions

NTHi strains 10P129H1 (*modA15*) and 84P36H1 (*modA18*) were isolated from COPD patients in Buffalo, USA, as part of a previous study^21^. The institutional review boards of the University at Buffalo and the Veterans Affairs Western New York Healthcare System approved collection of samples, as detailed previously^21^; study participants provided written informed consent before enrollment, as detailed previously^21^. All methods were performed in accordance with the relevant guidelines and regulations of the University at Buffalo and the Veterans Affairs Western New York Healthcare System, as detailed previously^21^. NTHi were grown in BHI broth (Oxoid) supplemented (sBHI) with hemin (1% v/v) and NAD (2 µg/ml), or on sBHI agar (as broth but with 1% w/v bacteriological agar; Oxoid). Liquid cultures were grown aerobically at 37 °C with shaking at 150 rpm. Plates were grown at 37 °C supplemented with 5% (v/v) CO_2_. Natural *modA15* and *modA18* ON and OFF variants in NTHi strains 10P129H1 and 84P36H1, respectively, were identified by fragment length analysis of the *modA* repeat tract of multiple single colonies using the fluorescently labeled (6-Carboxyfluorescein; FAM) forward primer Him1F (5′-FAM-ATGGCGGACAAAGCACCGAAGG-3′) and the reverse primer Him3 (5′-CAAAAAGCCGGTCAATTTCATCAAA-3′)^22^, and fragments were analyzed by the Australian Genome Research Facility (AGRF, Brisbane, Australia). Isolates containing ≥90% ON or OFF were considered to be natural ON or OFF respectively, as determined by fragment length analysis, and were used in subsequent studies. Fluorescently labelled fragments were measured using a 3130xl Genetic Analyser and GeneScan system (Applied Biosystems), and peaks analysed using Peakscanner version 1.0 (Applied Biosystems). We have previously described the *modA15* and *modA18* ON/OFF pairs^23^ and shown by Western blot that expression only occurs in the ON strain of each strain pair^23^.

### Analysis of *modA* allelic diversity in COPD isolates

Whole genome shot-gun sequencing had previously been carried out for all 269 NTHi strains isolated from COPD patients^21^. These genome sequences were used in a BLAST search using prototype *modA* alleles^20^, and the *modA* sequence isolated from each strain. Alignments were carried out using Clustal-W, and each allele manually checked in a nucleotide sequence alignment against the twenty-one known *modA* alleles. To compare the distribution of *modA* alleles in our COPD collection with the distribution of *modA* alleles in our previously published OM collection^19^, we used a one-tailed *z*-score test to compare individual *modA* allele proportions between the two populations.

### Preparation of outer membrane proteins (OMPs) from NTHi

NTHi *modA15 and modA18* ON/OFF pairs were grown in sBHI broth (50 ml) at 37 °C overnight with shaking at 100 rpm. Cells were pelleted at 4500 rpm for 15 mins at 4 °C, resuspended in 4 ml 10 mM HEPES-NaOH pH 7.5, and OMPs were prepared as detailed previously^24^. Briefly, cells were lysed by sonication, and debris pelleted as above. Sarkosyl was added to the clarified supernatant to a final concentration of 1%, and incubated at 25 °C for 30 mins. Supernatants were then centrifuged at 35,000 rpm for 90 mins. Pellets were resuspended in 10 mM HEPES-NaOH pH 7.5, sarkosyl added to a final concentration of 1%, and incubation and centrifugation steps repeated twice more. Final pellets containing the OMP-enriched fraction were resuspended in 100 μl of 10 mM HEPES-NaOH pH 7.5, and the protein concentration quantified using the BCA protein assay kit according to manufacturer’s instructions (Thermo Scientific). Each of the OMP preparations (5 µg) were run on the Novex Bis-Tris pre-cast gel system with MOPS running buffer according to the manufacturer’s instructions (Life Technologies). Ammoniacal silver staining was carried out to visualize proteins^25^ and observe if any proteins were differentially expressed due to *modA* phase-variation. Differences were identified visually, and confirmed by processing each band with imageJ using a band that was observed as the same intensity in the respective ON vs OFF lane (loading control; LC). Only differences that were confirmed by ImageJ as >2-fold intensity difference relative to the loading control were considered differentially expressed. Difference in intensity is presented as the fold difference ON vs OFF normalised to loading control.

### Single-Molecule, Real-Time (SMRT) sequencing and methylome analysis

We previously sequenced and submitted annotated genomes of NTHi strains 10P129H1 (*modA15*) and 84P36H1 (*modA18*)^23^. Briefly, DNA was sequenced at the Yale Center for Genome Analysis (YCGA) using the PacBio RS II platform with P6-C4 chemistry and a library size of 10 kb, with one strain per SMRT cell, and assembled *de novo* using the hierarchical genome assembly process (HGAP)^26^. Polishing for a pure-PacBio assembly was carried out using the Quiver algorithm^26^ from the SMRT Analysis software suite (version 2.3.0 – http://www.pacb.com/devnet/) with default parameters. Consensus sequences were submitted to NCBI for annotation with the Prokaryotic Genome Annotation Pipeline (PGAP), and annotated sequences submitted to GenBank (accession numbers CP029620 [strain 10P129H1] and CP029621 [strain 84P36H1]). Methylome analysis was carried out as described previously^27,28^ by YCGA. Each genome was sequenced to sufficient depth to allow methylome analysis of m6A methylation (cytosine methylation was not searched for). Coverage of each strain was as follows - strain 10P129H1 - ModA15 ON coverage of 393.5 fold, and ModA15 OFF coverage of 435.5 fold; strain 84P36H1 – ModA18 ON coverage of 435.5 fold, ModA18 OFF coverage of 380.0 fold.

### RNA Seq analysis

Triplicate biological replicates of total RNA were prepared using Trizol (Thermo Fisher) according to manufacturer’s instructions from mid-log cultures of NTHi *modA15* and *modA18* ON/OFF pairs (OD_600_ = 0.5) as previously used for *modA* ON vs OFF expression analysis^19^. RNA Seq data sets used have been recently announced^29^. RNA quality was assessed by AGRF using an Agilent Bioanalyser. All RNA preps had an RNA integrity number (RIN) of above 8.0, indicating high quality RNA. Libraries were prepared using the Illumina Ribo-Zero Gold protocol. Briefly, RNA was fragmented, and randomly primed first strand cDNA synthesis carried out using SuperScript II Reverse Transcriptase (Invitrogen) according to manufacturer’s protocols. Following second-strand cDNA synthesis, fragments were adenylated at the 3′ end, and polyT containing sequencing adapters ligated. Libraries were then amplified via PCR (13 cycles). Library quality was assessed using an Agilent Bioanalyser DNA 1000 chip. qPCR quantification was used to assess individual libraries before normalizing (2 nM) and pooling using the Illumina cBot system with TruSeq PE Cluster Kit v3 reagents. Sequencing (150 bp paired end runs) was performed on the Illumina NovaSeq system with TruSeq SBS Kit v3 reagents (average number of sequence reads for each triplicate sample is as follows – *modA15* ON - 37,726,839; *modA15* OFF - 32,023,168; *modA18* ON - 42,069,056; *modA18* OFF - 34,004,813). Sequence quality was assessed according to the standard protocols of AGRF. Unfiltered sequence reads were aligned against the respective reference genomes (CP029620 [10P129H1; *modA15*]; CP029621 [84P36H1; *modA18*]) using Bowtie2 aligner (v2.3.3.1) using standard settings. Default software settings were used throughout unless otherwise stated. Transcripts were assembled with Stringtie v1.3.3 (http://ccb.jhu.edu/software/stringtie/) utilising the reads alignment and reference annotation based assembly option. This methodology generated assemblies for known and potentially novel transcripts. The Gencode annotation containing both coding and non-coding annotation for each genome was used as a guide (http://www.gencodegenes.org/). Raw gene count values were analysed with edgeR (https://bioconductor.org/packages/release/ bioc/html/edgeR.html) to compute differential gene expression values. Counts were summarised at the gene level using the featureCounts v1.5.3 utility of the subread package (http://subread.sourceforge.net/). Gene expression differences between respective *modA* ON and OFF were expressed as logFC (log2-fold change of expression). Analysis generated logCPM values (average log count per million for the gene across all samples), F values (quasi-likelihood F-statistic for the gene across all samples), p-values for the test of statistically different expression, and the FDR (False discovery rate/Adjusted p-value for multiple hypothesis testing). Annotated genomes for each strain were used as the reference genome CP029620 (10P129H1; *modA15* ON/OFF pair) and CP029621 (84P36H1; *modA18* ON/OFF pair).

## Results and Discussion

### An NTHi collection isolated from COPD patients contains different proportions of modA alleles compared to those isolated from OM patients

**Figure 1:**
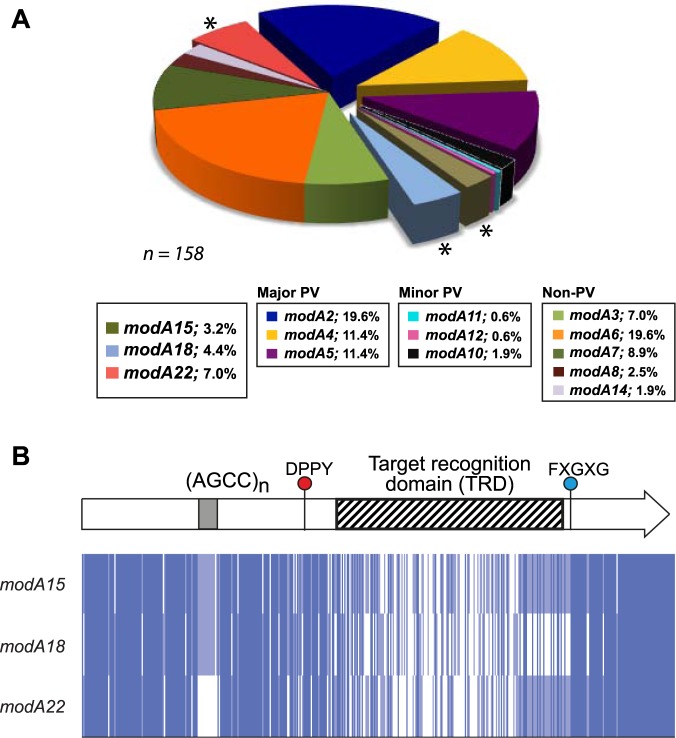
Distribution of the *modA* allele in a collection of NTHi isolated from patients with COPD. We analysed 269 unique strains taken from COPD patients and previously genome sequenced^21^ consisting of 67 cleared strains, and 101 persistent strain pairs (202 strains in total). BLAST search and sequence alignment for each strain was carried out in order to determine the *modA* allele present in each strain. We only analysed the 67 cleared strains, and the first isolate of each strain pair (n = 168). Of these 168 strains, 7 strains did not contain a *modA* allele, and 3 strains did not contain sufficient sequence to determine the *modA* allele present. (**A)** The *modA* allele distribution in 158 strains where we could unequivocally identify the *modA* allele sequence. *modA15*, *modA18*, and *modA22* are highlighted with a *. PV = phase-variable. The % of each *modA* allele present in the collection is given in the key; (**B)** sequence alignment of newly identified/studied *modA* alleles *modA15*, *modA18*, and *modA22* (newly identified as part of this study). Alignments were carried out using ClustalW, and visualized in JalView overview feature. Nucleotides are represented as vertical blue bars (dark blue >80% identity; light blue >50% identity; white <50% identity or gap.

Using sequenced genomes for 269 NTHi isolates from patients presenting with COPD^21^ we carried out BLAST analysis with our previously, well-defined *modA* allele sequences^19,20^ to determine the *modA* allele distribution within this population. The collection contains 168 independent strains consisting of 67 cleared strains and the first and last isolates of 101 persistent strains (n = 202) collected longitudinally over a fifteen year period (April 1994–March 2009)^21^. Cleared strains are defined as those isolated once at a single monthly visit to the clinic, then not isolated again on subsequent visits^21^. For *modA* allele distribution, we only counted the cleared strains (n = 67) and the first isolate of each persistent strain pair (n = 101), meaning we analysed the *modA* allele distribution in 168 unique strains. We could not detect any significant homology to any *modA* allele in seven of these strains, indicating that these isolates do not contain a *modA* gene. We have not observed this before in any other data sets, i.e., all previously sequenced NTHi always contain a *modA* gene. There was insufficient sequence coverage to accurately determine the *modA* allele present in 3 strains. Therefore 158 strains in total were analysed for their *modA* allele distribution. In order to determine if there is a different proportion of *modA* alleles associated with COPD isolates, we compared this collection to our previously characterised OM collection^19^. The majority (82.5%) of these isolates where collected in the USA^19,22,30,31^, with the remaining 17.5% from Finland^32^. Three major OM-associated phase-variable *modA* alleles, *modA2, 4*, and *5*, were all represented in COPD isolates at very similar levels to those seen in NTHi isolated from our previous studies^19^ (Fig. 1; *modA2* [19.6% COPD vs 17.2% OM], *modA4* [11.4% COPD vs 12.4% OM] and *modA5* ([11.4% COPD vs 9.5% OM]; *p* value of >0.05 for all three alleles using a one-tailed *z*-score test). However, the two other major *modA* alleles seen in children with OM, *modA9* and *modA10*^19^ were present in a much reduced proportion of strains in our COPD population; *modA9* was not present in any strain isolated from the COPD airway (vs 4.7% of OM isolates; a statistically significant *p* value of 0.0028 using a one-tailed *z*-score test), and *modA10* was present in only 3 COPD isolates (1.9%) despite being the second most prevalent phase-variable *modA* allele present in our OM isolate collection (15.3% OM; a statistically significant *p* value of <0.00001 using a one-tailed *z*-score test)^19^. This implies that the genes being differentially regulated by *modA9* and *modA10* provide an advantage, and are therefore selected for, in the OM middle ear, but not in COPD airways. It is even possible that the phasevarions controlled by *modA9* and *modA10* are disadvantageous in the context of COPD airways. Alternatively, and as a caveat to our statistical analysis, the different distribution of *modA* alleles in COPD and OM isolates could be due to geographic and/or temporal differences in the two strain collections: COPD isolates were collected at the Buffalo Veterans Affairs Medical Center, New York, USA, between 1994–2009^21^; OM strains comprise a number of different sub-collections collected in multiple locations across the globe, and have been detailed previously^19^. Therefore, the precise role, if any, of *modA9* and *modA10* in COPD would require extensive experimental confirmation, and this is hindered by the lack of any non-COPD isolates from the Buffalo area to determine if the lack of *modA9* in isolates taken from the COPD lung are due to selection against this allele in COPD, or due to lack of this allele in general in strains from this area.

During our analysis we characterised phasevarions controlled by two previously described *modA* alleles - *modA15* and *modA18 -* and discovered one new *modA* allele, which we have designated *modA22*. Although *modA15* and *modA18* had been identified previously^20^, their methylation specificity and the genes that they control, i.e., their respective phasevarions, had never been defined. Analysis of the collection showed that five strains contained *modA15* (3.16%) and seven strains contained *modA18* (4.43%). All *modA* genes in the study contain a AGCC_[n]_ DNA repeat tract of variable length, which mediates phase-variable expression. We picked prototype strains containing each allele - strain 10P129H1 contained *modA15* and strain 84P36H1 contained *modA18*. We have previously sequenced and annotated the genomes of these strains (Accession numbers CP029620 (strain 10P129H1) and CP029621 (strain 84P36H1)^23^. Fragment length analysis showed that the AGCC_[n]_ repeat tracts of these strains was phase-variable, and we were able to isolate ON and OFF enriched populations^23^.

The new *modA* allele identified in our COPD collection, which we have designated *modA22*, was not phase-variable. All strains containing a *modA22* allele (11 strains; 6.96%) contained three AGTC repeats in place of a variable AGCC_[n]_ repeat tract. Simple sequence repeat (SSR) tracts of this length phase-vary at very low rates^33–35^, and our own analysis showed only a single peak when carrying out fragment length analysis across this region of DNA, with no evidence of any sub-populations showing different SSR tract lengths (data not shown).

### ModA15 and ModA18 methylate different target sequences to currently characterised ModA alleles

We used methylome analysis in order to determine the methyltransferase specificity of ModA15 and ModA18. Using our previously described methodology and rationale^19,36^ we compared the methylomes from the ModA15 and ModA18 ON/OFF pairs to determine the specificity of each ModA methyltransferase. This analysis showed that ModA15 methylates the sequence 5′-G^(m6A)^**A**NTCNNCG-3′, and ModA18 methylates the sequence 5′-CTS^(m6A)^**A**GNNNNCG-3′ (Table 1). Both these sequences are unusual for Type III methyltransferases, as the majority recognise and methylate five base-pair non-palindromic sequences^37,38^. The ModA15 recognition sequence, 5′-G^(m6A)^**A**NTCNNCG-3′, occurs just 364 times in the genome of NTHi strain 10P129H1, and the ModA18 recognition sequence 5′-CTS^(m6A)^**A**GNNNNCG-3′ appears only 94 times in the genome of NTHi strain 84P36H1. Unlike other ModA alleles that methylate >95% of their recognition sites^19,36^, we could only detect methylation at 15.4% (56/364) of ModA15 sites, and at 48.9% (46/94) of ModA18 sites in the respective *modA* ON genomes. No methylation of these sequences was detected in the respective *modA* OFF genomes of each strain (Table 1). Our previous Western blot analysis of the ON/OFF pairs used for SMRT sequencing shows that ModA is expressed in each ON strain^23^ so the low methylation coverage was unexpected. However, these methyltransferases may recognise a hierarchy of sequences, with the identity of the non-specific bases (non-specific bases or ‘N’ refers to any of the four DNA bases A, C, G, or T) in each consensus sequence playing a role in the specificity/methylation at that particular site. This relaxed specificity has been observed before with ModA11 in *Neisseria meningitidis*^36^, with between 100% and 4.6% methylation detected at specific sites, dependent on the non-specific bases within the 5′-NCGY^(m6)^**A**GN-3′ consensus recognition sequence. In both ModA15 and Mod18 ON/OFF pairs we could detect Dam methylation at its characterised 5′-G^(m6A)^**A**TC-3′ site, but at only approximately 65% of all GATC sites in all four genomes (Table 1). We have previously seen at least 95% of GATC sites methylated in other genomes where we have carried out methylome analysis^19,36^. Perhaps the low level of methylation seen with both strains, including with Dam, is a result of either relaxed specificity, or a deficiency in the metabolism of these strains, but this would require extensive experimental confirmation, and is beyond the scope of the current study. An interesting follow up study could be to determine if competition with other methyltransferases, or other DNA binding proteins, exists at ModA15 and ModA18 sites to explain low levels of methylation, or if there is a general, as yet unknown, deficiency in ability to methylate in these strains.

**Table 1:** SMRT methylome data from *modA15* and *modA18* ON/OFF pairs.

Methylation site*	ModA15 ON	ModA15 OFF	ModA18 ON	ModA18 OFF	encoding gene (REBASE name)
5′-G^(m6A)^**A**NTCNNCG-3′	15.4% (56/364)	0%			DLJ98_09780 (M.Hin10P129H1I)
5′-CTS^(m6A)^**A**GNNNNCG-3′			48.9% (46/94)	0%	DLK00_02835 (M.Hin84P36H1I)
5′-G^(m6A)^**A**TC-3′	69.2%	67.6%	69.9%	68.2%	Dam DNA methyltransferase

*N is any base, S is G or C. Grey shading indicates that the methyltransferase gene is not present in this strain.

### Phase-variation of ModA15 and ModA18 results in outer-membrane protein expression differences

**Figure 2:**
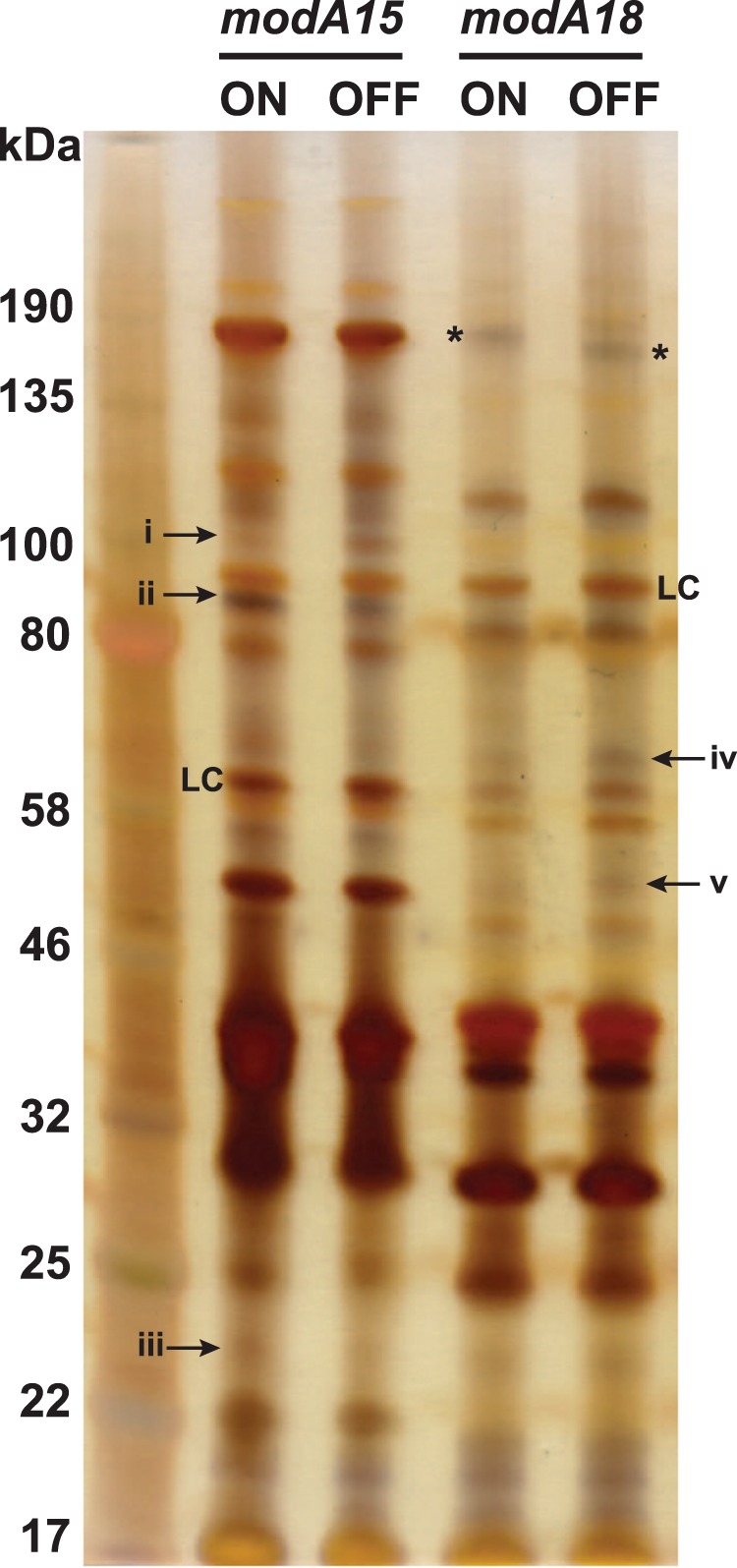
Silver stained protein gel showing outer-membrane protein (OMP) expression differences resulting from *modA15* and *modA18* phase-variation. OMPs were prepared using a sarkosyl OMP preparation protocol. 5 μg of each OMP fraction was separated on SDS PAGE and visualized using ammoniacal silver staining. Differences in protein expression between *modA15* ON vs OFF, and *modA18* ON vs OFF are highlighted with arrows. Differences were identified visually, and confirmed by processing each band with imageJ using a band that was observed as the same intensity in the respective ON vs OFF lane (loading control; LC). Only differences that were confirmed by ImageJ as >2-fold intensity difference ON vs OFF relative to the loading control are highlighted. Difference in intensity as fold difference ON vs OFF normalised to loading control. *modA15* ON vs *modA15* OFF: (i) 0.11 fold; (ii) 4.4 fold; (iii) 6.3 fold. *modA18* ON vs *modA18* OFF: (iv) 0.46 fold; (v) 0.40 fold. In addition, a difference in size of a protein of equivalent expression is highlighted with * in *modA18* ON vs OFF. The full gel image is provided in Supplementary Fig. 1.

In order to investigate if phase-variation of *modA15* and *modA18* resulted in differences in protein expression, we prepared outer-membrane protein (OMP) fractions from our *modA15* and *modA18* ON/OFF pairs. Following ammoniacal silver staining of 5 µg of each OMP preparation, clear differences in the expression level of several proteins is visible when comparing *modA15* ON vs OFF, and *modA18* ON vs OFF (Fig. 2). Differences in size of a number of comparable protein bands can also been seen, implying that *modA15* and *modA18* phase-variation leads to differences in expression of allelic variants of these proteins, or in differences in post-translational modifications of these proteins. As described in previously characterised *modA* phasevarions^19^, this demonstrates that phase-variation of *modA15* and *modA18* results in gene and protein expression differences, and that these *modA* alleles control phasevarions. Differences in expression of outer-membrane proteins are likely to influence the interaction of the bacterial population and the human host. As the outer-membrane of NTHi is the main interface with the host, this could have implications on pathobiology. Phase variation of OMPs could also impact vaccine development, as alterations in the expression level and/or modification state of proteins targeted by vaccines could render those vaccines ineffective if the target is no longer expressed or is modified so as to prevent recognition of epitopes.

### RNA Seq analysis reveals phase-variation of *modA15* and *modA18* results in whole cell gene expression differences

In order to determine the effects of *modA15* and *modA18* phase-variation on whole cell gene expression differences, we carried out whole cell RNA Seq analysis using a single ON/OFF pair of strains for each *modA* allele. RNA was prepared from the same cultures used to carry out Western blotting to show that each methyltransferase is only expressed in the ON strain, and confirmed by fragment length analysis, as described previously^23^. Our analysis showed that 4 genes were up-regulated and 40 genes down-regulated in *modA15* ON relative to OFF, and that 8 genes were up-regulated and 2 genes down-regulated in *modA18* ON relative to OFF (Table 2). Given the unusual recognition sequences of these two ModA alleles, and their rarity in the genomes of the strains containing them, the small number of differentially expressed genes is not surprising. A comparison of the genome sequence of each strain pair showed there were minimal differences between each strain pair except within the relevant *modA* AGCC_[n]_ repeat tract.

**Table 2:** RNA-Seq results from *modA15* and *modA18* ON/OFF pairs. Only genes with >2 fold expression differences are shown.

Locus tag	Protein product	Fold change (ON:OFF)	log_2_ fold change (ON:OFF)	logCPM	PValue	FDR
**UP in modA15 ON**						
DLJ98_09775	ResA15	7.13	2.83	9.06	2.48E-12	1.22E-09
DLJ98_09780	ModA15	3.18	1.67	8.47	4.77E-11	6.73E-09
DLJ98_05145	DNA binding transcriptional regulator Fis	2.35	1.23	7.34	2.54E-08	5.33E-07
DLJ98_08705	DNA polymerase 1	2.05	1.04	6.98	1.35E-08	3.55E-07
**DOWN in modA15 ON**						
DLJ98_05515	D-ribose pyranase	0.11	−3.24	5.71	3.90E-10	3.59E-08
DLJ98_05530	ribose ABC transporter substrate-binding protein RbsB	0.12	−3.11	8.52	3.18E-13	4.94E-10
DLJ98_07630	ornithine carbamoyltransferase	0.18	−2.46	10.64	3.15E-12	1.22E-09
DLJ98_07625	ArcC carbamate kinase	0.22	−2.21	10.59	8.78E-12	2.27E-09
DLJ98_04590	TbpA extracellular solute binding protein	0.22	−2.16	8.18	1.20E-11	2.66E-09
DLJ98_00210	DUF465 domain-containing protein	0.23	−2.12	7.92	2.76E-09	1.26E-07
DLJ98_03210	sodium/proline symporter PutP	0.23	−2.11	9.56	4.72E-11	6.73E-09
DLJ98_04585	ABC transporter substrate binding protein	0.24	−2.05	8.66	4.15E-12	1.29E-09
DLJ98_05485	acid phosphatase AphA	0.29	−1.81	11.77	6.72E-10	4.54E-08
DLJ98_09395	dithiol-disulfide isomerase	0.29	−1.79	5.08	2.66E-07	2.74E-06
DLJ98_06725	ketol-acid reductoisomerase	0.30	−1.74	10.70	3.10E-12	1.22E-09
DLJ98_04420	YjhT family mutarotase	0.31	−1.68	9.06	2.11E-11	4.10E-09
DLJ98_04410	sialic acid-binding protein	0.36	−1.48	11.85	4.16E-10	3.59E-08
DLJ98_02355	cold shock domain protein CspD	0.37	−1.44	9.38	3.45E-09	1.37E-07
DLJ98_01875	class II fumarate hydratase	0.37	−1.43	8.89	4.45E-11	6.73E-09
DLJ98_00385	NADP-dependent malic enzyme	0.37	−1.42	11.15	4.09E-10	3.59E-08
DLJ98_08875	universal stress protein UspA	0.39	−1.37	10.15	9.74E-09	2.75E-07
DLJ98_08840	galactose ABC transporter substrate-binding protein	0.39	−1.36	12.37	7.16E-10	4.63E-08
DLJ98_07615	Lrp/AsnC family transcriptional regulator	0.40	−1.33	5.47	2.66E-06	1.77E-05
DLJ98_03795	malate dehydrogenase	0.40	−1.32	8.84	6.57E-10	4.54E-08
DLJ98_06225	serine transporter	0.43	−1.23	4.41	4.35E-07	4.12E-06
DLJ98_06035	ribosome-associated translation inhibitor RaiA	0.44	−1.19	8.38	2.83E-08	5.77E-07
DLJ98_04600	ferric ABC transporter ATP-binding protein	0.45	−1.17	7.52	3.03E-10	3.29E-08
DLJ98_04425	isoprenylcysteine carboxylmethyltransferase family protein	0.45	−1.16	3.41	1.95E-05	8.71E-05
DLJ98_06760	glycerophosphodiester phosphodiesterase	0.45	−1.15	11.97	9.32E-11	1.21E-08
DLJ98_05535	ribokinase	0.45	−1.14	6.89	1.26E-08	3.44E-07
DLJ98_00010	autonomous glycyl radical cofactor GrcA	0.45	−1.14	10.22	4.87E-07	4.45E-06
DLJ98_09550	transporter	0.45	−1.14	9.10	6.39E-08	1.03E-06
DLJ98_07785	phospho-sugar mutase	0.46	−1.12	11.42	3.18E-10	3.29E-08
DLJ98_10115	phosphonate ABC transporter substrate-binding protein	0.46	−1.11	9.63	5.19E-09	1.75E-07
DLJ98_03455	uracil phosphoribosyltransferase	0.47	−1.10	9.58	1.55E-09	8.04E-08
DLJ98_05540	LacI family DNA-binding transcriptional regulator	0.48	−1.07	6.34	2.98E-09	1.28E-07
DLJ98_04415	C4-dicarboxylate ABC transporter permease	0.48	−1.07	10.06	3.31E-09	1.35E-07
DLJ98_04335	pyridoxal phosphate-dependent aminotransferase	0.48	−1.07	1.74	0.000137	0.000429
DLJ98_10260	TRAP transporter substrate-binding protein	0.48	−1.07	5.31	6.47E-08	1.04E-06
DLJ98_04915	NAD nucleotidase	0.48	−1.05	11.61	3.03E-10	3.29E-08
DLJ98_03460	uracil permease	0.49	−1.03	7.40	5.75E-10	4.25E-08
DLJ98_01765	hypothetical	0.50	−1.01	5.75	0.002699	0.005533
DLJ98_09605	polymerase	0.50	−1.01	9.38	5.30E-09	1.75E-07
DLJ98_04115	hypoxanthine phosphoribosyltransferase	0.50	−1.00	5.82	1.19E-07	1.61E-06
**UP in modA18 ON**						
DLK00_02840	ResA18	7.92	2.99	9.49	1.83E-11	2.98E-08
DLK00_02880	DmsB	4.34	2.12	5.54	3.03E-08	2.47E-05
DLK00_02885	DmsC	3.54	1.83	5.67	1.77E-06	0.00032
DLK00_02890	DmsD	2.90	1.54	3.96	1.65E-05	0.000958
DLK00_09770	flippase	2.51	1.33	10.48	0.173477	0.33405
DLK00_05505	hypothetical	2.13	1.09	4.85	3.55E-06	0.000414
DLK00_05510	hypothetical	2.07	1.05	4.66	2.15E-05	0.001002
DLK00_05500	regulatory protein GemA	2.00	0.98	5.23	5.94E-06	0.00051
**DOWN in modA18 ON**						
DLK00_10225	DUF935 domain-containing protein	0.50	−0.95	0.17	0.008281	0.045171
DLK00_07345	Lic3A/Lic3B	0.31	−1.70	5.77	2.47E-06	0.000352

In the ModA15 phasevarion, the DNA binding transcriptional regulator Fis [DLJ98_05145] was up-regulated in ON relative to OFF. Therefore, in addition to the direct regulatory effects of ModA15 methylation, i.e., regulation of genes by differential methylation of individual gene promoter regions, the differential regulation of Fis could be thought of as indirect gene regulation by *modA* phase-variation^39^ (regulation of a regulator – the gene expression changes resulting from Fis being differentially regulated are indirectly due to ModA15, as *modA15* phase-variation regulates the expression of the regulator Fis). Fis has been shown to modulate virulence gene expression in a number of bacterial pathogens, including *Salmonella enterica* and *Dickeya dadantii*^40^, so differential regulation of Fis by ModA15 could play a key role in NTHi pathobiology. The transcriptional repressor LacI (DLJ98_05540) is differentially regulated in the ModA15 phasevarion, showing a 2-fold decrease in expression in ModA15 ON. Lower expression of LacI in ModA15 ON may impart more metabolic flexibility *in vivo* due to increased expression of genes involved in carbon metabolism. A number of metabolic genes are down-regulated in the ModA15 phasevarion when ModA15 is ON; these include genes involved in nucleotide metabolism (e.g., D-ribose pyranase [DLJ98_05515], hypoxanthine phosphoribosyltransferase [DLJ98_04115]) central carbon metabolism (C4-dicarboxylate ABC transporter permease [DLJ98_04415], malate dehydrogenase [DLJ98_03795]) and phosphate metabolism (acid phosphatase AphA [DLJ98_05485], phosphonate ABC transporter substrate-binding protein [DLJ98_10115]). This suggests that a sub-population of cells where ModA15 is OFF may be better equipped to colonise different host niches compared to sub-populations of cells where ModA15 is ON, and indicates that dynamic interplay between ModA15 ON and ModA15 OFF sub-populations could occur during colonisation and disease.

A number of genes regulated in the ModA18 phasevarion could have effects on NTHi pathobiology; for example, ModA18 ON shows increased expression of the regulatory protein GemA (DLK00_05500), that is widespread in the Pasteurellaceae, but has an as yet undefined regulon. The anaerobic dimethyl sulphoxide reductase (DmsBCD; DLK00_02880 - DLK00_02890) is also up-regulated in ModA18 ON. Dimethyl sulphoxide reductase has been shown to be important in virulence in *Actinobacillus pleuropneumoniae*^41^.

Our demonstration of the presence of two new phase-variable *modA* alleles controlling phasevarions in NTHi adds an extra level of complexity to vaccine development in this organism. The identification of what could be considered COPD-associated *modA* alleles, *modA15* and *modA18*, means that characterisation of the complete phasevarions regulated by these two alleles could aid development of more effective treatment options for COPD patients colonised by NTHi, as well as directing and informing overall vaccine development against NTHi.

## Supplementary information

Supplementary Information
