# Supplementary material for: Non-typeable Haemophilus influenzae isolates from patients with chronic obstructive pulmonary disease contain new phase-variable modA methyltransferase alleles controlling phasevarions
Source: Sci Rep. 2019 Nov 4;9:15963. doi: 10.1038/s41598-019-52429-6 (PMC6828955; doi:10.1038/s41598-019-52429-6)

Supplementary Information for:

Non-typeable Haemophilus influenzae isolates from patients with chronic obstructive pulmonary disease contain new phase-variable modA methyltransferase alleles controlling phasevarions

John M. Atack, Timothy F. Murphy, Melinda M. Pettigrew, Kate L. Seib and Michael P. Jennings

Supplementary Figure 1 -
full gel image from which Figure 2 is taken. Area used for figure 2 is highlighted with a dashed line

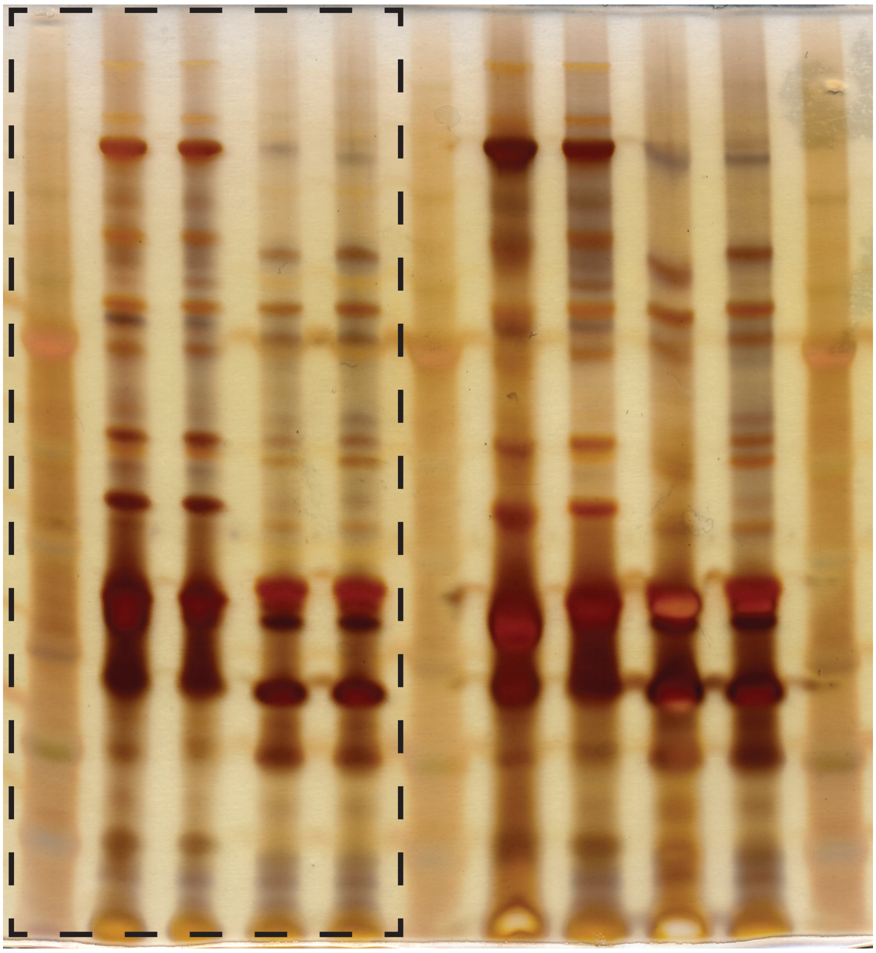

Supplement: Supplementary file 1 — Supplementary Information [file 41598_2019_52429_MOESM1_ESM.pdf]
